# Global view of the RAF-MEK-ERK module and its immediate downstream effectors

**DOI:** 10.1038/s41598-019-47245-x

**Published:** 2019-07-26

**Authors:** Cristina C. Santini, James Longden, Erwin M. Schoof, Craig D. Simpson, Grace R. Jeschke, Pau Creixell, Jinho Kim, Xuewei Wu, Benjamin E. Turk, Neal Rosen, Poulikos I. Poulikakos, Rune Linding

**Affiliations:** 1 Technical University of Denmark (DTU), Kgs. Lyngby, 2800 Denmark; 2 Biotech Research & Innovation Centre, University of Copenhagen, Copenhagen, 2200 Denmark; 3 Department of Pharmacology, Yale University, New Haven, 06520 USA; 4 Koch Institute for Integrative Cancer Research, Massachusetts Institute of Technology, Cambridge, USA; 5 Samsung Genome Institute, Samsung medical Center, Seoul, 06351 South Korea; 6 Icahn School of Medicine at Mount Sinai, New York, 10029-5674 USA; 7 Memorial Sloan Kettering Cancer Center, New York, 10065 USA; 8 Institute of Biology, Humboldt-Universität zu Berlin, Berlin, 10115 Germany; 9 Present Address: Celgene Institute Translational Research Europe (CITRE), Seville, E-41092 Spain

**Keywords:** Cell signalling, Mass spectrometry, Cancer

## Abstract

Small molecule inhibitors of BRAF and MEK have proven effective at inhibiting tumor growth in melanoma patients, however this efficacy is limited due to the almost universal development of drug resistance. To provide advanced insight into the signaling responses that occur following kinase inhibition we have performed quantitative (phospho)-proteomics of human melanoma cells treated with either dabrafenib, a BRAF inhibitor; trametinib, a MEK inhibitor or SCH772984, an ERK inhibitor. Over nine experiments we identified 7827 class I phosphorylation sites on 4960 proteins. This included 54 phosphorylation sites that were significantly down-modulated after exposure to all three inhibitors, 34 of which have not been previously reported. Functional analysis of these novel ERK targets identified roles for them in GTPase activity and regulation, apoptosis and cell-cell adhesion. Comparison of the results presented here with previously reported phosphorylation sites downstream of ERK showed a limited degree of overlap suggesting that ERK signaling responses may be highly cell line and cue specific. In addition we identified 26 phosphorylation sites that were only responsive to dabrafenib. We provide further orthogonal experimental evidence for 3 of these sites in human embryonic kidney cells over-expressing BRAF as well as further computational insights using KinomeXplorer. The validated phosphorylation sites were found to be involved in actin regulation, which has been proposed as a novel mechanism for inhibiting resistance development. These results would suggest that the linearity of the BRAF-MEK-ERK module is at least context dependent.

## Introduction

The ERK signaling network plays a central role in multiple cellular processes, including proliferation, differentiation, development, learning survival and apoptosis^1^. Signalling is usually initiated by the small G protein RAS which transmits the signal to RAF kinases. In turn RAF kinases phosphorylate MAPK/ERK kinases (MEK) which phosphorylate ERK kinases^2^. In order for this relatively simple network to affect the many diverse, and conflicting, cellular processes it controls, sophisticated regulatory mechanisms are required. Abnormalities in this regulation have been shown to occur in many cancers. In fact RAF family kinases (ARAF, BRAF and RAF1) were among the first oncoproteins to be described approximately 30 years ago^3^. It is now known that BRAF mutations occur in a wide-range of cancers, particularly those with RAS mutations^4^. The highest frequency of BRAF mutations are found in malignant melanoma; approximately 50% of melanomas have BRAF mutations and approximately 90% of those are BRAF^V600E ^^5^. The BRAF^V600E^ mutation results in the constitutive activity of BRAF deregulating the activation of its downstream effectors, including MEK and ERK, observations that have led to the development of small molecule inhibitors that target these kinases. These inhibitors are initially very effective at removing tumor burden, however they are rarely curative due to the rapid onset of drug resistance. Even though the mechanisms of resistance to therapy are diverse for each tumor, in more than two thirds of BRAF^V600E^ positive tumors, MAPK signaling networks are reactivated^6^.

**Figure 1:**
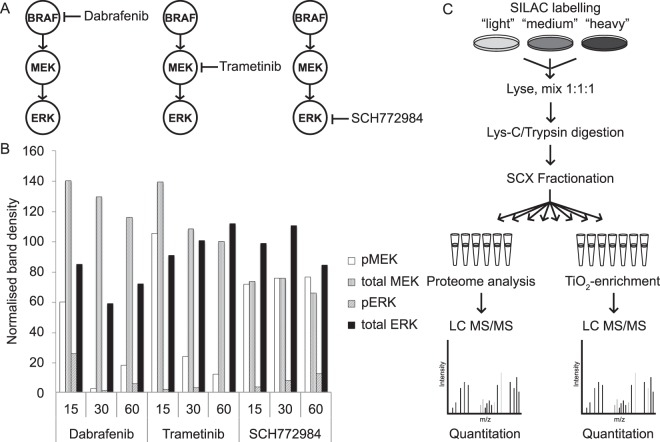
Method overview. (**A**) The BRAF, MEK, ERK kinase module was inhibited in 3 independent experiments using small molecule inhibitors specific to either BRAF (dabrafenib), MEK (trametinib) and ERK (SCH772984). (**B**) Analysis of the change in phosphorylation of MEK and ERK over time following drug treatment was assessed by western blot. Quantification of the density of the observed bands (shown in Supplementary Fig. S1) was performed in ImageJ. Data was normalized relative to the DMSO treated cells. (**C**) Cells were labelled with isotopomeric versions of the amino acids argenine and lysine. Following drug treatment cells were lysed and proteins extracted, digested and fractionated. Phosphorylated peptides were enriched using titanium dioxide prior to mass spectrometry analysis.

Although phosphoproteomic studies identifying targets of ERK have recently been published the overlap between them has been relatively low^7^ and they have invariably focused on just one or two inhibitors. Given that it is well known that kinase signaling networks can be multivariate in nature^8^ we propose that in order to fully understand the complex responses of kinases to small molecule inhibition in an aberrant signalling context the systematic perturbation of the entire network is required. To that end, we here describe the global phospho-proteomic and proteomic analysis of A375 cells, harboring a mutated form of the BRAF kinase (V600E), treated with the BRAF inhibitor dabrafenib, the MEK inhibitor trametinib and the ERK inhibitor SCH772984 (Fig. 1A). By inhibiting each member of the signaling network independently we provide unique insight into the flow of information through these proteins identifying novel effectors of BRAF and ERK. This proteomic analysis was combined with exome sequencing, an approach now commonly referred to as proteogenomics^9^. By constructing genome-specific spectra databases more proteins and phosphorylation sites can be identified due to the inclusion of mutated proteins and peptides, thus facilitating more accurate and comprehensive modeling of cancer signaling networks.

## Results

### Perturbation of the RAF-MEK-ERK signaling cascade

Distinct populations of cells were treated with either dabrafenib, trametinib or SCH772984 at EC100 concentration (500 nM for dabrafenib, 100 nM for trametinib and 500 nM for SCH772984) for 30 minutes (further details can be found in the Experimental Procedures). The time-point used was selected following western blot analysis of protein samples from A375 cells treated for 15 minutes, 30 minutes and 1 hour with dabrafenib, trametinib and SCH772984 (Figs 1B and S1). After treatment with dabrafenib we observed a significant decrease in phosphorylated MEK and ERK at all time points. After trametinib treatment we observed a significant decrease in phosphorylated MEK at 30 minutes and 1 hour, while there was a significant decrease in phosphorylated ERK at all time-points. After treatment with SCH772984 we observed no significant change in the amount of phosphorylated MEK but did observe a significant decrease in ERK phosphorylation at all time points. We therefore selected the 30 minute time-point as the most optimal for further phosphoproteomic analyses as significant inhibition of phosphorylation was observed following treatment with all three inhibitors.

### Global phosphoproteomic analysis of the effects of RAF, MEK and ERK inhibitors on signaling

Modulation of cell signaling following inhibitor treatment was quantified using global phosphoproteomics and stable isotope labelling by amino acids in cell culture (SILAC) (Fig. 1C). In order to confirm that the A375 cells were SILAC labelled, argenine and lysine incorporation, as well as proline conversion rates were monitored by determining the ratio of labelled peptides to unlabelled peptides in a test sample. We found that 98.6% of arginine, and 98.7% of lysine, amino acids were SILAC labelled whilst proline conversion was 1.34% (Supplementary Fig. S2); given the extremely high level of SILAC incorporation it was determined that the potential for erroneous measurements due to inadequate labelling would be negligible^10^. SILAC ‘light’-labeled cells (Lys 0, Arg 0), treated with a vehicle control (dimethyl sulfoxide, (DMSO)), ‘medium’-labelled cells (Lys 4, Arg 6), a ‘super-SILAC’^11^ mix of cells from all treatment conditions, along with the ‘heavy’-labeled (Lys8, Arg 10) drug treated cells were lysed in denaturing conditions (modified RIPA buffer with protease/phosphatase inhibitors) to prevent spurious kinase or phosphate activities. Cell lysates were quantified and mixed in a 1:1:1 ratio for each SILAC experiment and digested with Lys-C and trypsin. Peptides were then separated into fractions using strong cation exchange chromatography (SCX); seven SCX fractions were phospho-peptide enriched using titanium dioxide beads to enable the identification of occupied serine, threonine and tyrosine phosphorylation sites. Proteome analysis was performed using six SCX fractions in order to obtain global quantitative data on the relative protein abundances and to normalize phosphorylation levels to their respective protein levels. Samples were analysed by LC-MS/MS on a Q-Exactive Orbitrap mass spectrometer in biological triplicate; data was found to be consistent across the replicates with Pearson’s correlation coefficients ranging from 0.54 to 0.7 (Supplementary Fig. S2).

### Identification of modulated phosphorylated peptides

**Figure 2:**
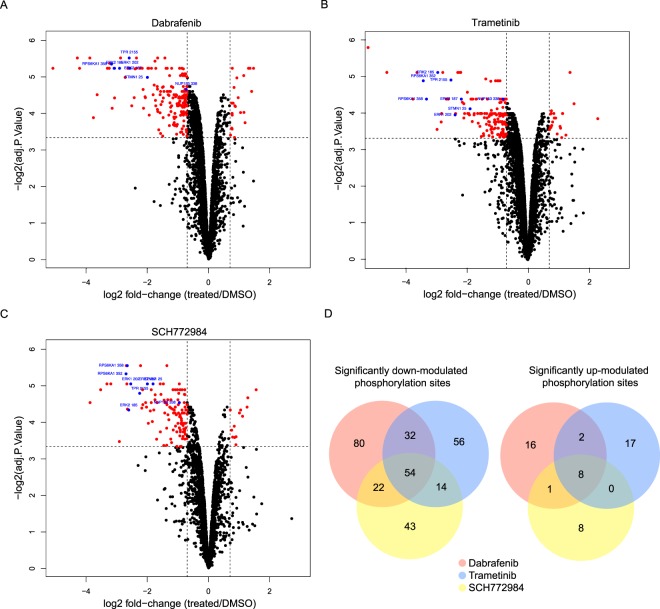
All identified phosphorylation sites observed following treatment with (**A**) dabrafenib, **(B**) trametinib or (**C**) SCH772984. Significantly modulated phosphorylation sites are shown in red, known effectors of ERK used to validate the dataset (as listed in Table S2) are shown in blue. (**D**) Number of down-modulated phosphorylation sites (limma FDR < 0.1 and log_2_FC < −0.7) and up-modulated phosphorylation sites (limma FDR < 0.1 and log_2_FC > 0.7) observed in each tested condition.

Raw files were processed using MaxQuant 1.5.0.2^12^ selecting only the class I phosphorylation sites^13^ (Class I phosphorylation sites were defined by a localization probability of 0.75 and a probability localization score difference greater than or equal to 5). Quantified ratios of median phophosite intensities were then calculated by dividing the intensity of the heavy samples by the intensity of the light samples. Similarly, phosphorylation site intensities were normalized to proteome by dividing the phosphorylation site ratio by the ratio of heavy to light protein intensities. This normalization allowed the direct comparison of phosphorylated peptides following treatment by different inhibitors. These phospho/proteome ratios were then log_2_ scaled such that negative ratios indicate a down-modulation of phosphorylation sites and positive ratios indicate an up-modulation. For example, a phosphorylation site with a log_2_(phosphorylation site H/L:protein H/L) ratio (log_2_FC) of −1 would have a 50% reduction in phosphorylation sites following inhibitor treatment compared to the DMSO control treatment, a fold-change of 0.5. Following this method we identified 7827 class I phosphorylation sites from at least one of the 9 conducted experiments (Supplementary Table S1). In order to further classify the modulated phosphorylation sites, as either up or down modulated, we ranked phosphorylation sites according to their log_2_FC ratio and the limma adjusted P value false-discovery rate (limma FDR). We established a ratio threshold by searching the observed phosphorylation sites for known effectors of ERK^14–17^, identifying phosphorylation sites on STMN1, TPR, RPS6KA1 and NUP153 all consistently down-modulated after inhibitor treatment as well as phosphorylation sites on the ERK proteins themselves (Supplementary Table S2). From this analysis we determined that known effectors of ERK had at least a −0.7 log_2_FC and a limma FDR less than 0.1 in our assay. We therefore used these thresholds to identify other significantly modulated phosphorylation sites respective to inhibitor treatment (Fig. 2A–C). Globally, this analysis identified 301 down-modulated phosphorylation sites and 52 up-modulated phosphorylation sites (Fig. 2D). Only those phosphorylation sites that were significantly down-modulated in the presence of all three inhibitors were considered downstream effectors of ERK.

### Identified effectors of ERK

**Figure 3:**
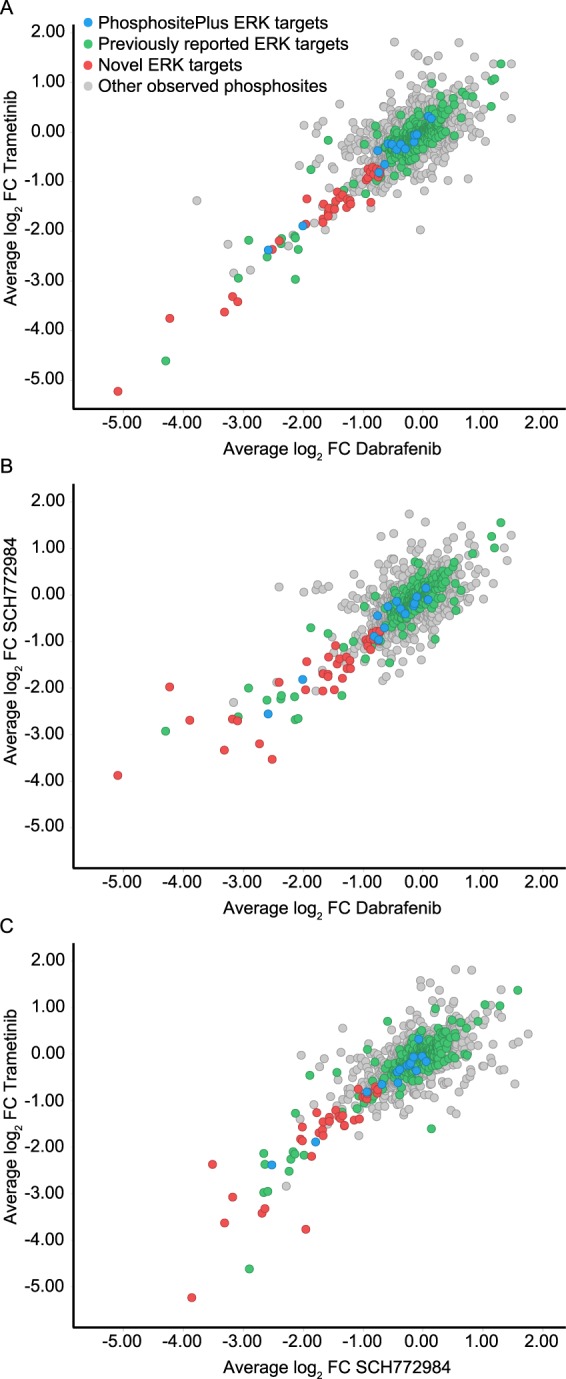
Effect of known and novel ERK targets on the phosphorylation of BRAF, MEK and ERK. (**A**) Fold change in phosphorylation following inhibition with dabrafenib (a BRAF inhibitor) and trametinib (a MEK inhibitor). **(B**) Fold change in phosphorylation following inhibition with dabrafenib and SCH772984 (an ERK inhibitor). (**C**) Fold change in phosphorylation following inhibition with trametinib and SCH772984. In all cases ERK targets listed in PhosphositePlus are shown in blue, ERK targets reported by similar phosphoproteomic studies are shown in green. The 34 novel ERK targets passing fold change and false discovery rate thresholds in this assay are shown in red.

We identified 54 phosphorylation sites down-modulated after treatment with dabrafenib, trametinib and SCH772984 (Fig. 2D and Supplementary Table S3). As these represent the most likely ERK effectors we compared this list to previously reported studies using human cancer cell lines^7,18–21^ and ERK1 and ERK2 substrates reported in the PhosphoSitePlus database^22^ (Fig. 3). Of the 54 modulated phosphorylation sites identified, 34 have not been previously reported. We therefore sought to validate these 54 phosphorylation sites using computational and experimental means. We used KinomeXplorer^23^, a tool for predicting kinase-substrate relationships using sequence-based classifiers of linear motifs, to generate substrate predictions for ERK1 and ERK2. Of the 54 identified, 11 phosphorylation sites were found to have ERK1 or ERK2 as the highest KinomeXplorer prediction (Supplementary Table S4). In addition to this computational validation we also screened HEK293 cells over-expressing FLAG-tagged ERK1 or ERK2 in an affinity purification mass spectrometry experiment to identify protein-protein interactions^24^. Any proteins physically interacting with ERK1 or ERK2 could then be identified by capturing the FLAG-tag using anti-FLAG beads. The success of the affinity purification was validated by quantifying the intensity of ERK1 or ERK2; as expected we found a significant enrichment of ERK1 and ERK2 in their respective samples both with a z-score in excess of 5000. Of the 41 proteins (from the 54 phosphorylation sites) identified in the A375 studies 13 could be validated as physically interacting with ERK1 and/or ERK2 using affinity purification (Supplementary Table S5).

Analysis of changes occurring at the level of the proteome, following the same assessment criteria described above for the phosphorylation sites, found that no proteins were significantly regulated after treatment with all inhibitors (Supplementary Table S6).

### Differentially modulated phosphorylation sites

**Figure 4:**
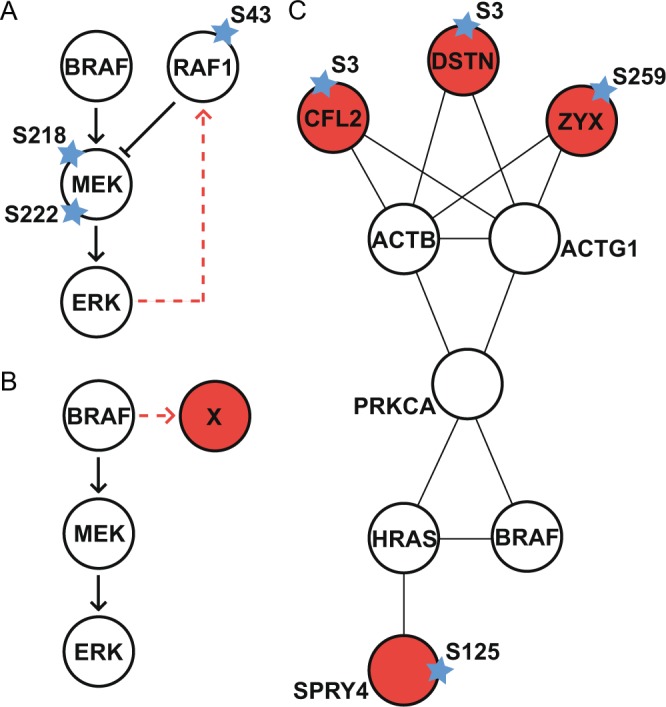
Phosphorylation patterns observed in this study. (**A**) Known phosphorylation sites observed in this study that did not pass the thresholds set. MEK S222 was significantly down-modulated after MEK inhibition, but as expected, was not significantly modulated after ERK inhibition. RAF1 S43, from the known inhibitory ERK feedback loop^25^, was found to be significantly modulated after trametinib and SCH772984 treatment but not dabrafenib. (**B**) We identified 26 phosphorylation sites that were significantly modulated following BRAF inhibition, but not after MEK and ERK and could thus be direct targets of BRAF. Of the 26 potential BRAF targets 3 could be validated by experimental means and 1 by computational means. The simplest STRING network illustrating their currently known interaction targets and BRAF is shown in (**C**).

While the combined analysis of the effect of the three inhibitors facilitated the reliable determination of downstream effectors of ERK we also investigated the signal propagation following inhibition with each of the three tested inhibitors individually. We identified 301 phosphorylation sites that were differentially down-modulated by the three inhibitors, these were either known modulation sites upstream of ERK (such as the MEK sites, S218 and S222), known effectors of ERK that failed to pass the modulation threshold set, or BRAF/MEK/ERK-specific effectors (Fig. 4). The first two cases are illustrated in Fig. 4A; MEK S218 was downmodulated after BRAF and MEK inhibition but was not observed in all samples and thus did not pass the modulation threshold, MEK S222 was significantly down-modulated after MEK inhibition, but as expected, was not significantly modulated after ERK inhibition. The known ERK feedback mechanism via the inhibitory site RAF1 S43^25^ also failed to pass the modulation threshold set for all 3 independent experiments; this site was deemed significantly down-modulated after MEK and ERK inhibition (FDR < 0.1 and log_2_FC < −0.7), but was identified in only one of the biological replicates after BRAF inhibition (log_2_FC < −0.7) (Supplementary Table S1).

To determine the nature of the BRAF/MEK/ERK-specific effectors we selected the most significantly modulated phosphorylation sites (limma FDR < 0.05 and log_2_FC < −0.7) that were differentially modulated after treatment with dabrafenib, trametinib or SCH772984 alone (Supplementary Table S7). This identified 30 phosphorylation sites, the majority of which were modulated after treatment with dabrafenib (Fig. 4B). To further validate these phosphorylation sites we performed additional *in silico* and *in vitro* validation. As there was no public motif for BRAF one was generated using position-specific scoring matrices of FLAG-tagged BRAF isolated from mammalian cells (Supplementary Fig. S3). We found that BRAF did not have absolute specificity at any position but had a preference for Leucine and Methionine residues at the −3 position, hydrophobic residues at the +1 position and aromatic residues at the +2 position. Following the integration of this novel linear motif into NetPhorest/KinomeXplorer we found that, of the BRAF-specific effectors identified in this study, SPRY4 S125 was also predicted to be a BRAF substrate (Supplementary Table S8). In addition, the BRAF-specific effectors, CFL2 S3, DSTN S3 and ZYX S259 were also found to have significantly modulated phosphorylation sites in a HEK293 cell line over expressing BRAF (Supplementary Table S7).

## Discussion

In this study we identified 54 effectors of ERK, just over half of which could be successfully validated either experimentally, using physical interaction assays with ERK1/2, or computationally, from their linear motif. Despite this, we observed a relatively poor overlap with known ERK effectors, which is not unique to this investigation. By combining our data with previously reported studies^7,18–21^ we found a total of 1515 unique phosphorylation sites (on 707 unique proteins) reported to be ERK effectors (Supplementary Table S9). It would seem likely that this is due to the complexity of the module and may suggest signaling responses which are multivariate in nature, dependent on the context of the model and cue given. In order to derive more functional insight from the novel phosphorylation sites observed in this study we imported the list of proteins into DAVID^26,27^. These previously unidentified phosphorylated proteins were found to have functional roles in GTPase activity and regulation, apoptosis and cell-cell adhesion, amongst others (Supplementary Table S10 and Supplementary Fig. S4). Given that these are known, albeit in some cases relatively understudied, areas of ERK signaling^28–30^ combined with the fact that there was a significant overlap of both computationally predicted substrates and direct interactors of ERK, identified in a drug independent manner, it would seem likely that the phosphorylation sites identified here are novel ERK effectors.

Whilst we identified significant numbers of novel phosphorylation sites we did not observe any significantly modulated proteins. This is perhaps unsurprising given the relatively short treatment time used in this assay. We did however find that APOB and THBS1 were significantly regulated after treatment with dabrafenib and that both proteins also decreased in concentration after treatment with trametinib and SCH772984, albeit not significantly. APOB is a lipoprotein whose overproduction is a characteristic of insulin resistance, which can lead to type 2 diabetes. This overproduction has previously been shown to be inhibited by ERK activation^31^. Similarly THBS1 has been shown to be involved in epithelial-to-mesenchymal transition in melanoma and the development of drug resistance, both via the tumor cells themselves^32^ and also by protective mechanisms in melanoma-associated fibroblasts^33^. These data support the observations made in this study and indicate a potential role for these proteins as diabetes and cancer therapeutic targets, respectively.

If the BRAF, MEK, ERK module is linear as suggested, then it should be expected that modulation of phosphorylation sites after treatment with BRAF and/or MEK inhibitors would also result in the modulation of these sites after ERK inhibition. Thus observations of differentially modulated phosphorylation sites must therefore either be erroneous, possibly caused by off-target effects of the inhibitors, or real, indicating that the network is not linear. ‘Pathway analysis’ of the phosphorylated proteins identified following treatment with dabrafenib, trametinib or SCH772984 was performed using DAVID (Supplementary Fig. S4). We found significant overlap between the function of the three inhibitors centered around signal transduction, GTPase activity, tissue development, apoptosis and cell adhesion. Following BRAF inhibition by dabrafenib we also observed phosphorylation of proteins involved in cytoskeletal organization which is, at least in part, due to the validatable phosphorylation sites identified in our study as BRAF effectors (CFL2, DSTN and ZYX). STRING^34^ analysis of protein-protein interactions linking CFL2, DSTN and ZYK to BRAF showed that all three interact with actin (Fig. 4C) an interesting observation given that it has recently been shown that prolonged exposure to BRAF inhibitors results in changes in cell shape and that inhibiting these changes may overcome drug resistance^35^. Our results would thus suggest CFL2, DSTN and/or ZYK modulation as a mechanism for this regulation of actin. SPRY4, predicted to be a direct target of BRAF, is a member of the sprouty family of proteins that are well known as evolutionary conserved modulators of the MAPK/ERK module^36^. They are known to be deregulated in cancer and have been shown to play a role in the development, progression and metastasis of tumors, as well as the initial adaptive response to BRAF inhibitors^37^. Recently it has been shown that SPRY4 is a transcriptional target of BRAF^V600E ^^38^, however the link between this transcriptional regulation and phosphorylation is currently unexplored. Given that the experimental validation for these phosphorylation sites was performed in the absence of drug, we can conclude that these were not drug-specific observations. It should also be noted that the phosphorylation of DSTN by BRAF has been reported before^7,17^. Taken together, these data would suggest that BRAF kinase can recognize peptides, and thus potentially have a larger substrate space than previously thought. This would support the notion that the RAF-MEK-ERK module may, like many other signaling systems, rely on non-linear dynamic interactions depending on context.

## Experimental Procedures

### Cell culture

A375 (ATCC, CRL-1619) cells were SILAC labelled over 6 passages such that the incorporation level was above 95%. Cells were routinely passaged in T75 flasks and expanded into 15 cm dishes for mass spectrometry experiments. Cells were serum starved for 24 hours once they had grown to 80% confluency in the dishes. Serum was then added back to the cells for 24 hours prior to treatment with one of the three tested compounds. Each treatment condition was repeated in triplicate with an associated DMSO control.

### Drug treatments

Dabrafenib (Selleckchem, S2807), trametinib (Selleckchem, S2673) and SCH772984 (Selleckchem, S7101) were diluted in DMSO to 500 μM, 100 μM and 500 μM concentrations respectively. Compounds were then aliquoted and stored at −80 °C. For cell treatment compounds were diluted 1/1000 in tissue culture medium and incubated with the cells for 30 minutes.

### Sample preparation

Sample preparation and TiO2 enrichment of phosphorylated peptides methods were based upon the protocol described in Schoof and Linding^39^. Cells were washed twice with 2 ml of ice-cold phosphate buffered saline before the addition of 1 ml of RIPA lysis buffer (50 mM Trizma hydrochloride (ph 7.5), 150 mM NaCl, 1% v/v IGEPAL CA-630, 0.5% w/v sodium deoxycholate, 1 mM EDTA, 5 mM B-glycerophosphate, 5 mM NaF, 1 mM Na3VO4, 1 Roche cOmplete, EDTA-free protease inhibitor cocktail per 10 ml). Cells were then sonicated in three 10 second bursts with an amplitude of 40% and then centrifuged at 4400 g for 20 minutes at 4 °C. Cells and all buffers were maintained at 4 °C throughout the lysis procedure. Following centrifugation the supernatant was decanted into a clean tube and the pellet discarded, 40 ml of acetone was then added to the supernatant and incubated at −20 °C overnight.

The following morning samples were centrifuged at 2000 g for 5 minutes at 4 °C, the supernatant was then discarded and the precipitate dissolved in 6 ml of urea buffer (6 M Urea, 2 M Thiourea, 10 mM HEPES pH 8). The sample was incubated at room temperature on a rotating platform until all protein had dissolved, if the precipitate did not dissolve supernatant was aspirated and additional urea buffer was added to facilitate this step. Protein concentration was then determined using the Qubit Protein Assay Kit (ThermoFisher Scientific), according to the manufacturers instructions. Samples were then mixed such that the resulting solution contained equal amounts (8 mg) of each of the ‘heavy’, ‘medium’ and ‘light’ SILAC labeled samples. 1 mM Dithiothritol was then added to this mixture and incubated for 1 hour on a rotating platform at room temperature. Chloroacetamide was then added at 1 in 100 dilution and incubated for 1 hour on a rotating platform at room temperature. Following this Lys-C protease (Wako Chemical Corporation, 129-02541) was added to the sample at a ratio of 1:200 (protease:substrate) and incubated for 4 hours on a rotating platform at room temperature. Samples were then diluted 4-fold in 50 mM ammonium bicarbonate (pH 8) prior to the addition of trypsin (Sigma-Aldrich, T6567) at a ratio of 1:200 (protease:substrate). Samples were then incubated overnight on a rotating platform at room temperature.

The following morning trifluoroacetic acid was added to the sample to give a 2% final concentration, the pH of the sample was then checked to confirm that it was below 2.5, with additional trifluoroacetic acid added as required. The sample was then centrifuged at 2000 g for 5 minutes and the supernatant decanted into a clean tube before being added to a 55–105 μm Sep-Pak C18 cartridge (Waters, WAT020515) that had been prewashed with 5 ml of acetonitrile and two 4 ml washes of 0.1% trifluoroacetic acid. After sample loading the cartridge was washed three times with 6 ml of 0.1% trifluoroacetic acid. Sample was then eluted in two 2 ml volumes of 60% acetonitrile, 0.1% trifluoroacetic acid and 2 ml of 80% acetonitrile, 0.1% trifluoroacetic acid. In all cases pressure was applied to the Sep-Pak such that flow rate was even and no greater than 0.5 ml per minute.

### TiO_2_ enrichment of phosphorylated peptides

Samples eluted from the Sep-Pak were then fractionated using strong cation exchange chromatography generating 32 fractions. Fractions 1 to 6, 7 to 11, 17 to 19, 20 to 25, 26 to 27 and 28 to 32 were pooled and, along with the individual fractions 12 to 16, 1.5 mg of titanium dioxide beads (GL Sciences, 5020–75000) in 6 μl DHB buffer (20 mg/ml 2,5-dihydroxybenzoic acid, 5% trifluoroacetic acid, 30% acetonitrile) were added. Samples were mixed with the titanium beads for 30 minutes on a rotating platform before being centrifuged at 2000 g for 5 minutes. The supernatant from fractions 1 to 6 and 7 to 11, fractions 12 to 14 and fractions 15 to 16 were decanted into a new tube and an additional 1.5 mg of titanium dioxide beads added and mixed for 30 minutes on a rotating platform. The supernatant from fractions 1 to 6 and 7 to 11 were decanted into a new tube with an additional 1.5 mg of titanium dioxide beads and mixed again for 30 minutes on a rotating platform. In total this generated 15 titanium bead samples. Following mixing supernatant was discarded and the titanium beads resuspended in 100 μl of 5 mM potassium dihydrogen phosphate, 30% acetonitrile, 350 mM KCl, pH 2.7 (with trifluoroacetic acid).

Samples were then centrifuged at 2000 g for 5 minutes, the supernatant aspirated and the beads resuspended in 100 μl of 40% acetonitrile, 0.25% acetic acid, 0.5% trifluoroacetic acid. Samples were again centrifuged at 2000 g for 5 minutes, supernatant discarded and the beads resuspended in 50 μl of 80% acetonitrile, 0.5% acetic acid. Suspended beads were transferred to a C8 stage tip and centrifuged at 1000 g for 30 seconds and repeated until all liquid had passed through the stage tip. Samples were then eluted from the beads into a pcr microplate using 20 μl of 5% ammonia and 20 μl of 10% ammonia, 25% acetonitrile with the eluent from stage tips 3 and 4, 5 and 6, 7 and 8 and 10 and 11 mixed to give a total of 11 samples. Volumes were normalised by the addition of 40 μl of acidification buffer (1% trifluoroacetic acid, 5% acetonitrile) to wells containing eluent from only one stage tip. Plates were then spun in a centrifugal evaporator until 5–10 μl of buffer remained, approximately 65 minutes.

For proteome analysis six samples were collected; 10 μl of sample prior to strong cation exchange fractionation, 10 μl from the fraction 1–6 pool, 10 μl from the fraction 7–11 pool, 10 μl from fractions 12 to 15 (2.5 μl from each), 10 μl from fraction 16 and the fraction 17–19 pool (5 μl from each) and 10 μl from the fraction 20–25, 26–27 and 28–32 pools (3.5 μl from each).

C18 stage tips were prepared by the addition of 20 μl of methanol centrifuged through the tip at 1000 g for 30 seconds. Stage tips were then washed with 20 μl 80% acetonitrile, 0.1% formic acid, 1000 g for 30 seconds and 20 μl of 3% acetonitrile, 1% trifluoroacetic acid, twice, again at 1000 g for 30 seconds. Samples were resuspended in acidification buffer, 20 μl for those containing a single eluent and 40 μl for pooled eluents. Plates were centrifuged for 1 minute to condense the sample, which was then transferred to the stage tip. Stage tips were centrifuged for 30 seconds at 1000 g and repeated until all sample had passed through the stage tip. Samples were the washed by the addition of 20 μl of 0.1% formic acid to the stage tips, before centrifugation at 1000 g for 30 seconds. This wash was then repeated a second time. Samples were then eluted from the stage tips into a pcr microplate using 20 μl of 80% acetonitrile, 0.1% formic acid, twice, for pooled samples and 40 μl of 80% acetonitrile, 0.1% formic acid, twice, for single samples. Eluent from stage tips 2 and 8, 3 and 4, 6 and 7 and 9 and 10 were mixed to give a total of 7 samples. Plates were then spun in a centrifugal evaporator for 35 minutes before the addition of 4 μl of 40% acetonitrile, 0.25% acetic acid, 0.5% trifluoroacetic acid. Plates were then centrifuged for 1 minute to condense the sample and analysed on a ThermoFisher Scientific Q Exactive mass spectrometer.

### Affinity purification

HEK293 cells were transfected with FLAG-tagged ERK1, ERK2 and control (red fluorescent protein) protein using the Flp-In T-Rex system (ThermoFisher Scientific. Gene expression was induced with tetracyclin for 72 hours prior to affinity purification sample preparation according to the protocol described in Lambert *et al*.^40^ with some minor alterations. Cells were washed with 4 ml of PBS before being scraped off the dish into 1 ml PBS using a rubber spatula. Cells from two 150 mm plates were pelleted by centrifugation, the supernatant removed and the pellet weighed before being frozen on dry ice and kept at −80 °C until ready to be used (pellet weights for samples used in this assay were 287, 302 and 338 mg for ERK1 triplicate repeats, 288, 284 and 377 mg for ERK2 and 282, 292 and 364 mg for control samples). Cells were lysed by resuspension in 1:4 (pellet weight/volume) ratio of lysis buffer (50 mM HEPES-NaOH (pH 8.0), 100 mM KCl, 2 mM EDTA, 0.2% NP40, 10% glycerol, 5 mM β-glycerophosphate, 5 mM sodium fluoride, 1 mM sodium orthovanadate and protease inhibitor cocktail (Sigma-Aldrich, 000000004693159001, 1 tablet/10 ml) followed by two freeze/thaw cycles and sonication at 20% amplitude for two 10 second bursts with a 3 mm probe. Samples were split into two and half was treated with 1 μl benzonase (250 units/μl) for 1 hour (4 °C on a rotator). Fractions were then recombined and the resulting cell extract was then clarified by centrifugation at 4500 RCF for 20 min (4 °C) before transferring the supernatant to a fresh tube. Affinity purifications were performed by incubating the cleared lysate with 25 μl of pre-washed magnetic M2 anti-FLAG beads (Sigma-Aldrich) for 2 hours at 4 °C on a rotator. Beads were then washed four times with 1 ml of 20 mM Tris-HCl pH 8 2 mM CaCl_2_ before being centrifuged (500 g for 1 minute) and any residual liquid removed.

For mass spectrometry analysis, samples underwent on-bead digestion. Beads were first resuspended in 20 μl of Tris-HCl buffer (pH 8) prior to the addition of 1 μg of Lys-C protease (Wako Chemical Corporation, 129–02541). Cells were incubated with the Lys-C for 3 hours on a rotator. Following this, 1 μg of trypsin (Sigma-Aldrich, T6567) was added to the sample and incubated for approximately 16 hours on a rotator at room temperature. The following morning, 1.2 μl of 50% formic acid was added to the samples to stop digestion and 20 μl of acidification buffer added to each sample. C18 stage tips were prepared by the addition of 20 μl of methanol centrifuged through the tip at 400 g for 35 seconds. Stage tips were then washed with 20 μl 80% acetonitrile, 0.1% formic acid, 400 g for 35 seconds, and 250 μl of 3% acetonitrile, 1% trifluoroacetic acid at 600 g for 1 minute. Samples were resuspended in 20 μl of acidification buffer and transferred to the stage tip. Stage tips were centrifuged for 1 minute at 600 g. Samples were then washed by the addition of 100 μl of 0.1% formic acid to the stage tips, centrifuged through at 800 g for 1 minute. This wash was then repeated a second time. Samples were then eluted from the stage tips into a pcr microplate using 40 μl of buffer B. Plates were then spun in a centrifugal evaporator for 65 minutes and analysed on a ThermoFisher Scientific Q Exactive mass spectrometer.

### Mass spectrometry

Approximately 1 μg of peptides from each proteome or phospho-proteome sample was loaded onto a 50 cm C18 EasySpray column (ThermoFisher Scientific, ES803) using an Easy-nLC 1000 (ThermoFisher Scientific) with the column oven set at 45°C and a flow rate of 250 nl/min. Phospho-proteome samples were eluted over a 250 minute gradient ranging from 5% to 50% acetonitrile. The Q Exactive was run in DDA-MS2 top10 method with full MS spectra collected at a resolution of 70,000, a scan range of 300 to 1750 m/z and an AGC target of 3 × 10^6^ or 20 ms maximum injection time. MS2 spectra were obtained at a resolution of 17,500 and an AGC target of 1 × 10^6^ or 80 ms maximum injection time. Dynamic exclusion was set to 20 seconds and ions with a charge state less than 2, or unknown, were excluded. For proteome analyses gradient time was decreased to 240 minutes, maximum MS injection time was decreased to 60 ms and dynamic exclusion was increased to 45 seconds, all other settings were identical.

### Mass spectrometry searches

Raw files were processed using MaxQuant 1.5.0.2 with all searches conducted using an A375-specific database, where all protein sequence variants were included in addition to the reference, Ensemble version 68 human FASTA, sequences (see FASTA file generation for details). Methionine oxidation, protein N-terminal acetylation and serine/threonine/tyrosine phosphorylation were set as variable modifications and cysteine carbamidomethylation was set as a fixed modification. False discovery rates were set to 1% and the ‘match between runs’ functionality was activated. The medium labeled super SILAC control was included in order to maximize SILAC ratio coverage, it was included in all searches but was not utilized for further analysis.

### FASTA file generation

A375 cells were grown to 80% confluence in T75 flasks. DNA was extracted from the cells using a Qiagen QIAamp DNA mini kit according to the manufacturers instructions. Sample was then sent to Beckman Coulter Genomics (now GENEWIZ) for sequencing. Target enrichment was performed using SureSelect whole exome version 5 and sequencing was performed using an Illumina system at 2 × 100 base pair reads, 89x average coverage with a minimum of 10x coverage at 95% of the sample.

Reads were filtered with Trimmomatic^41^ using the following parameters: headcrop = 3, minlen = 30, trailing = 3. Trimmed reads were aligned to the hg19 reference genome using the Burows-Wheeler Alignment tool^42^ before the application of the Genome Analysis Toolkit^43^ base quality score recalibration, indel realignment, duplicate removal, and SNP and INDEL discovery and genotyping across all samples simultaneously using standard hard filtering parameters according to Genome Analysis Toolkit best practices recommendations^44^. The Ensembl Variant Predictor^45^ was used to predict the effect of the mutations on the protein sequence and those passing a high sensitivity filter (QD ≤ 1.5, FS ≥ 60, MQ ≥ 40, MQRankSum ≤ −12.5, ReadPosRankSum ≤ −8 and DP ≥ 5 per sample on average) were added to the search FASTA file.

### Statistical analysis

Phosphorylation sites were assessed for significance using the limma FDR, a moderated t-test implemented in the limma package for Bioconductor (http://www.bioconductor.org). Significance p-values obtained were corrected for multiple testing by the false-discovery method^46^ and deemed significant at an FDR threshold of 0.1. Functional enrichment analysis was performed using DAVID with a background list comprised of the 4960 proteins identified in the mass spectrometry experiments. The resulting Gene Ontology terms were visualized using REVIGO^47^.

### Western blotting

4–12% Bis-Tris gels were transferred to PVDF transfer membranes and blocked for 1 hour with PBS-Tween containing 5% (w/v) milk powder. Primary antibodies were mouse anti-MEK (4694, Cell Signaling), mouse anti-ERK (9107, Cell Signaling), rabbit anti-RSK1/RSK2/RSK3 (9355, Cell Signaling), rabbit anti-phospho-MEK1/2 (Ser217/221, 9154, Cell Signaling) and rabbit anti-phospho-p44/42 MAPK (Thr202/Tyr204, 9101, Cell Signaling). Primary antibodies were added to membranes and incubated overnight at 4 °C at 1:1000, except for the anti-ERK antibody, which was used at 1:2000. Secondary antibodies were horseradish peroxidase-conjugated sheep anti-mouse (ab6808, Abcam) and horseradish peroxidase-conjugated goat anti-rabbit (ab6721, Abcam) immunoglobulin G (both used at 1:2000). Quantification was performed using ImageJ with the Analyze/Gels tool used to firstly define each lane. Plot Lanes was then used to create density plots of each designated lane. The Line and Hand tools were then used to remove background noise and calculate the area under the peak respectively. All data points were then expressed relative to the DMSO control.

### Peptide array analysis of BRAF phosphorylation site specificity

BRAF specificity was analyzed using an arrayed positional scanning peptide library as previously described^48^. Briefly, the library consisted of 180 peptide mixtures having the general sequence Y-A-x-x-x-x-x-S/T-x-x-x-x-A-G-K-K(biotin), where “x” indicates an equimolar mixture of the 17 amino acids excluding Cys, Ser and Thr. Each mixture had one “x” position fixed as one of the 20 amino acids. Peptide mixtures (50 µM) were incubated for 2 h at 30 °C with FLAG-BRAF-V600E (affinity purified from transiently transfected HEK293T cells as described) in a reaction buffer containing 50 mM HEPES, pH 7.4, 5 mM MgCl_2_, 5 mM MnCl_2_, 1 mM DTT, 0.1% Tween 20, and 50 µM [γ-^33^P]ATP (50 µCi/ml). Aliquots (200 nl) of each reaction were then transferred to a streptavidin-coated membrane (Promega), which was washed, dried, and exposed to a phosphor storage screen as described. Radiolabel incorporation into each peptide was quantified using QuantityOne software (BioRad). Quantified data were normalized to an average value of unity within each peptide position and log_2_ transformed. The heat map (generated in Microsoft Excel) shows data averaged from 2 independent assays.

## Supplementary information

Supplementary text and figures

Supplementary table 1

Supplementary table 2

Supplementary table 3

Supplementary table 4

Supplementary table 5

Supplementary table 6

Supplementary table 7

Supplementary table 8

Supplementary table 9

Supplementary table 10

## Data Availability

The mass spectrometry proteomics data have been deposited to the ProteomeXchange Consortium via the PRIDE partner repository with the dataset identifier PXD013923. The exome sequencing data have been deposited to the Sequence Read Archive with the accession PRJNA544116. All other data needed to evaluate the conclusions in the paper are present in the paper or the supplementary materials.
