# Supplementary material for: Global view of the RAF-MEK-ERK module and its immediate downstream effectors
Source: Sci Rep. 2019 Jul 26;9:10865. doi: 10.1038/s41598-019-47245-x (PMC6659682; doi:10.1038/s41598-019-47245-x)

Global view of the RAF-MEK-ERK module and its immediate downstream effectors

Cristina C. Santini[1,9†], James Longden[2†], Erwin M. Schoof[1], Craig D. Simpson[2], Grace R. Jeschke[3], Pau Creixell[4], Jinho Kim[5], Xuewei Wu[6], Benjamin E. Turk[3], Neal Rosen[7], Poulikos I. Poulikakos[6], Rune Linding[1,2,8*]

1 Technical University of Denmark (DTU), Kgs. Lyngby, DK-2800, Denmark

2 Biotech Research & Innovation Centre, University of Copenhagen, Copenhagen, DK-2200, Denmark

3 Department of Pharmacology, Yale University, New Haven, 06520, USA

4 Koch Institute for Integrative Cancer Research, Massachusetts Institute of Technology, Cambridge, USA

5 Samsung Genome Institute, Samsung medical Center, Seoul, 06351, South korea

6 Icahn School of Medicine at Mount Sinai, New York, 10029-5674, USA

7 Memorial Sloan Kettering Cancer Center, New York, 10065, USA

8 Institute of Biology, Humboldt-Universität zu Berlin, Berlin, 10115, Germany

Current address:

9 Celgene Institute Translational Research Europe (CITRE), Seville E-41092, Spain

[†] These authors contributed equally

Correspondence: linding@lindinglab.org (R.L).

Supplementary Materials

Supplementary Figure S1. Western Blot analysis of the effect of dabrafenib, trametinib and SCH772984 on phosphorylated MEK and ERK. All experiments were performed at the same time with cell lysates prepared for each condition and then split equally between the 5 gels. Total RSK was used as a sample processing control.

Supplementary Figure S2. SILAC labeling efficiency of A375 cells (A) and reproducibility of the triplicate repeats for the dabrafenib, trametinib and SCH772984 treated A375 cells (B).

Supplementary Figure S3. Position scanning peptide library array illustrating the BRAF motif.

Supplementary Figure S4. Visualization of the pathway analysis conducted using DAVID for significantly modulated phosphorylated proteins observed following treatment with all 3 inhibitors (A), dabrafenib alone (B), trametinib alone (C) or SCH772984 alone (D).

Table S1. Class I phosphorylation sites identified in the mass spectrometry experiments.

Table S2. Modulation of known effectors of ERK used to validate the observed dataset.

Table S3. Phosphorylation sites significantly down-modulated after treatment with all three inhibitors.

Table S4. Identified phosphorylation sites predicted to be ERK targets by KinomeXplorer.

Table S5. Identified proteins that were found to directly interact with ERK.

Table S6. Proteins identified in the mass spectrometry experiments.

Table S7. Differentially modulated high confidence phosphorylation sites.

Table S8. KinomeXplorer predictions for phosphorylation sites differentially modulated after treatment with dabrafenib.

Table S9. Known ERK effectors previously reported in the literature and novel effectors identified in this study.

Table S10. Functional analysis of novel ERK targets identified in this study.

Supplementary Figure S1

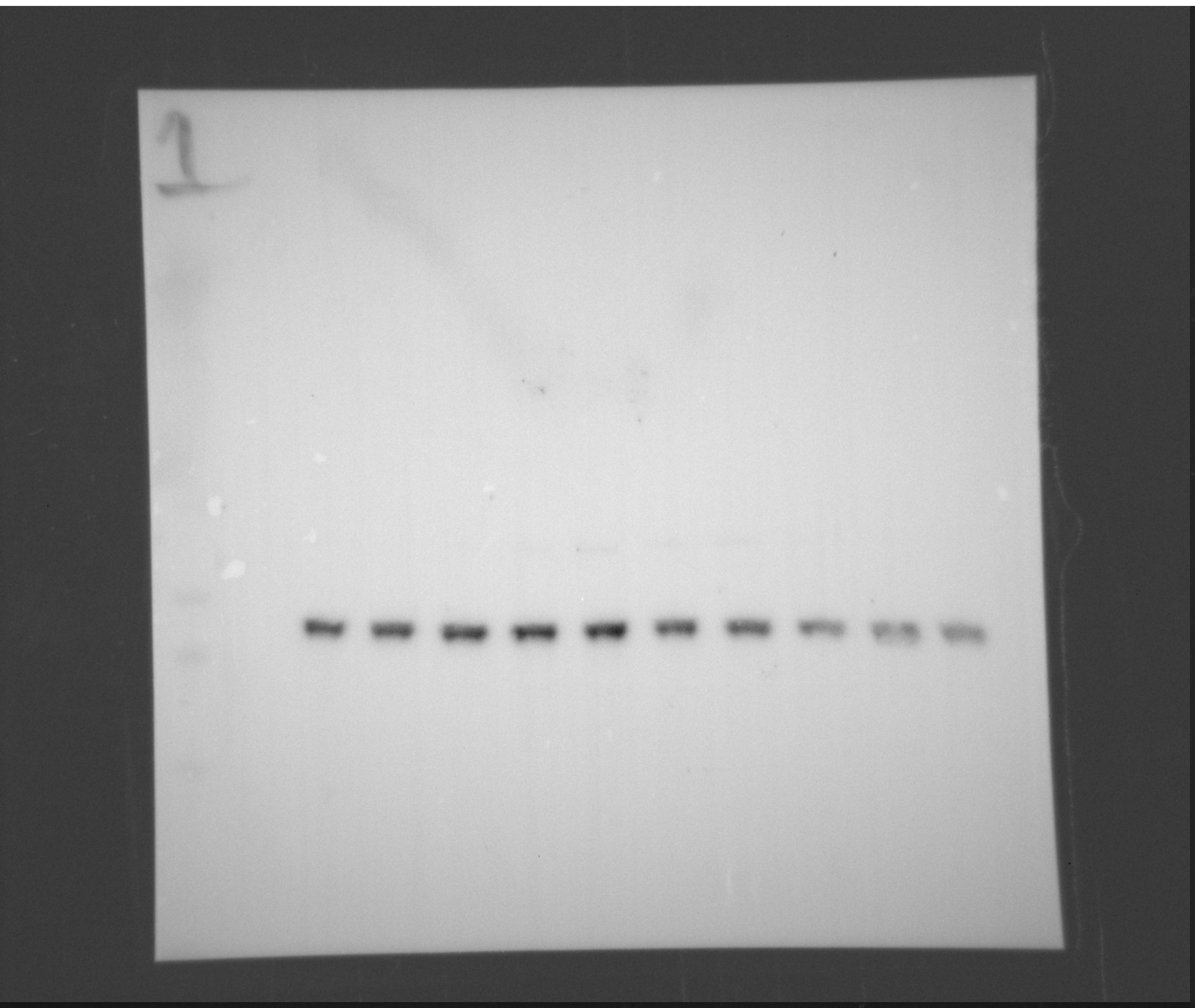

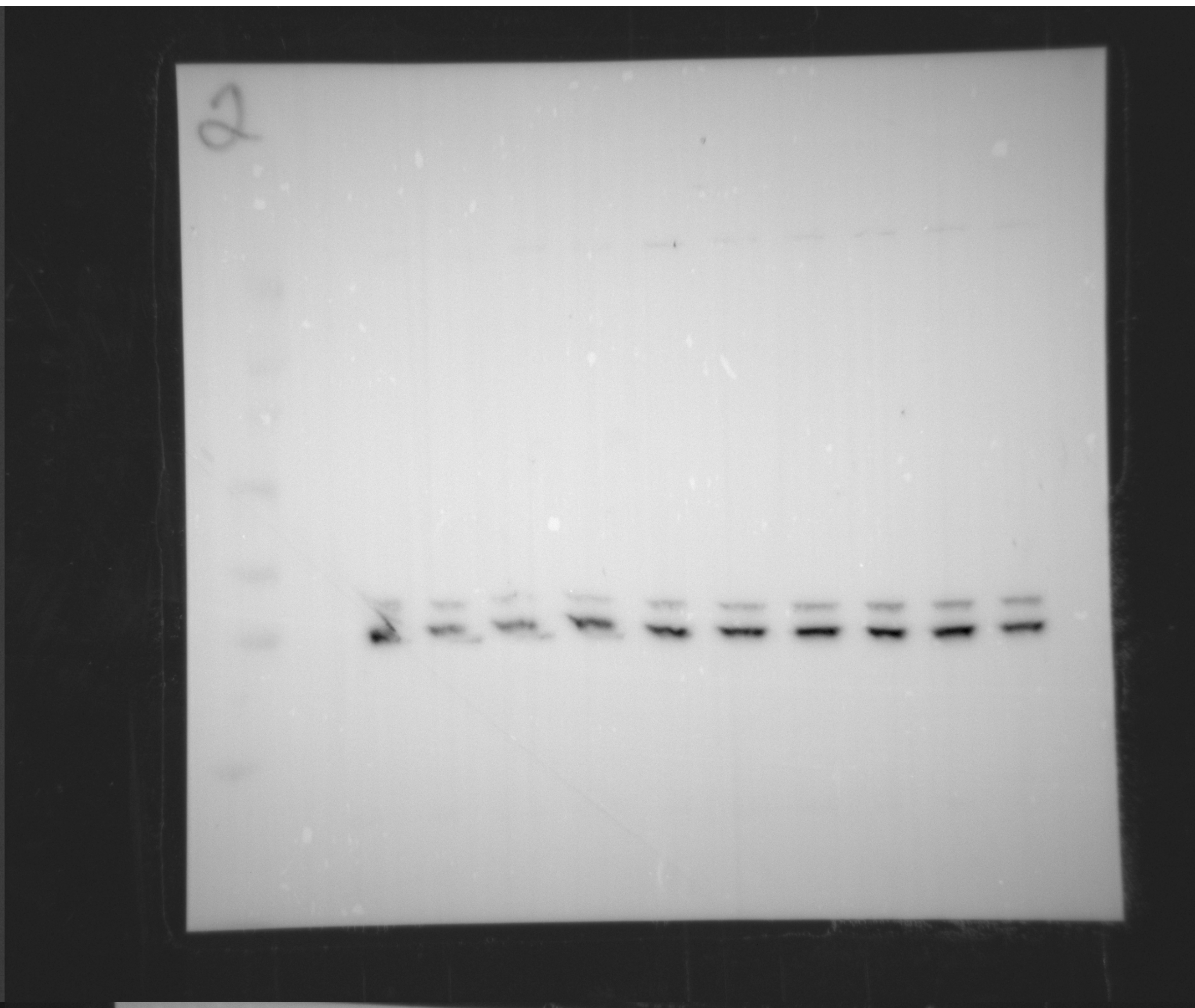

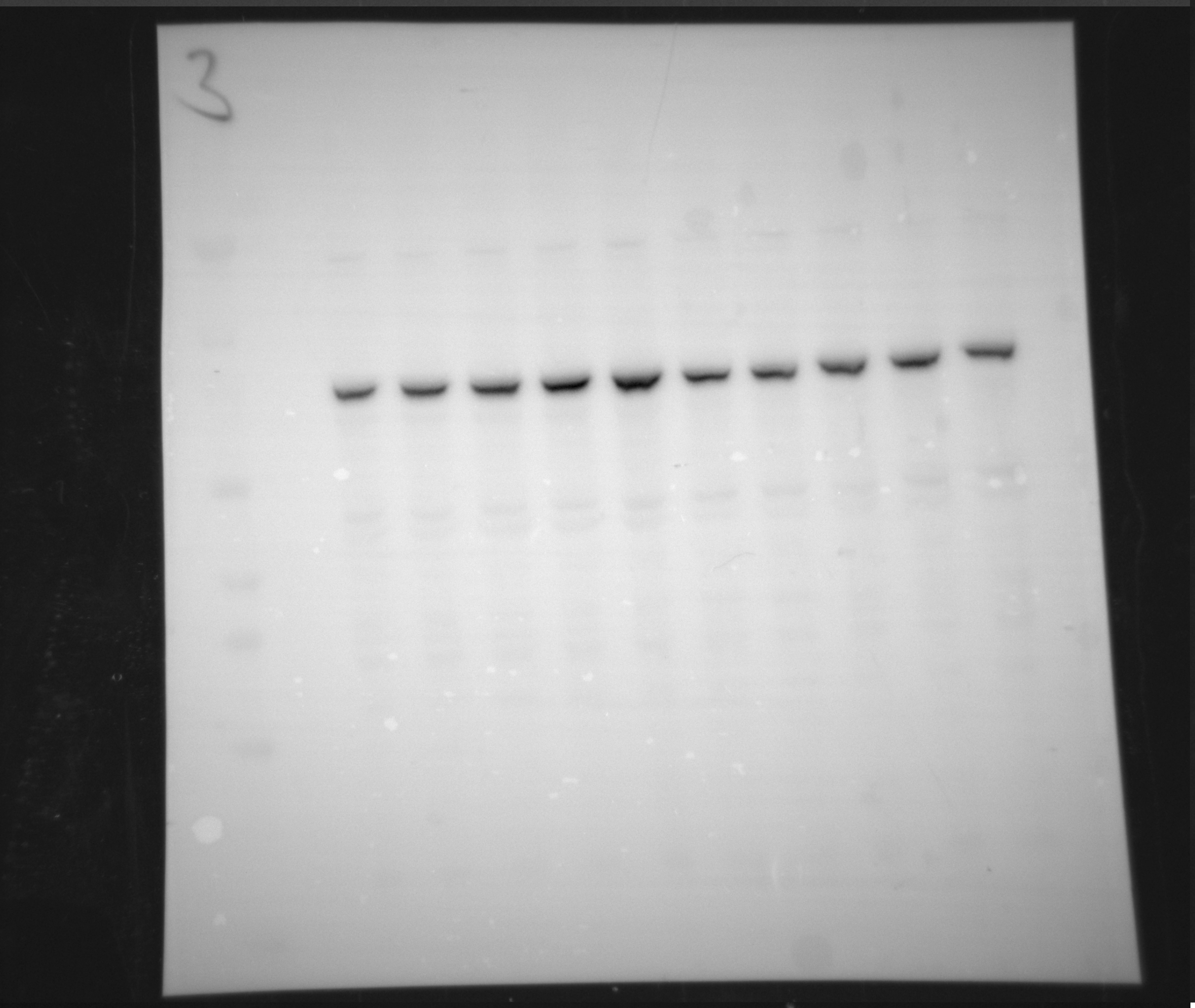

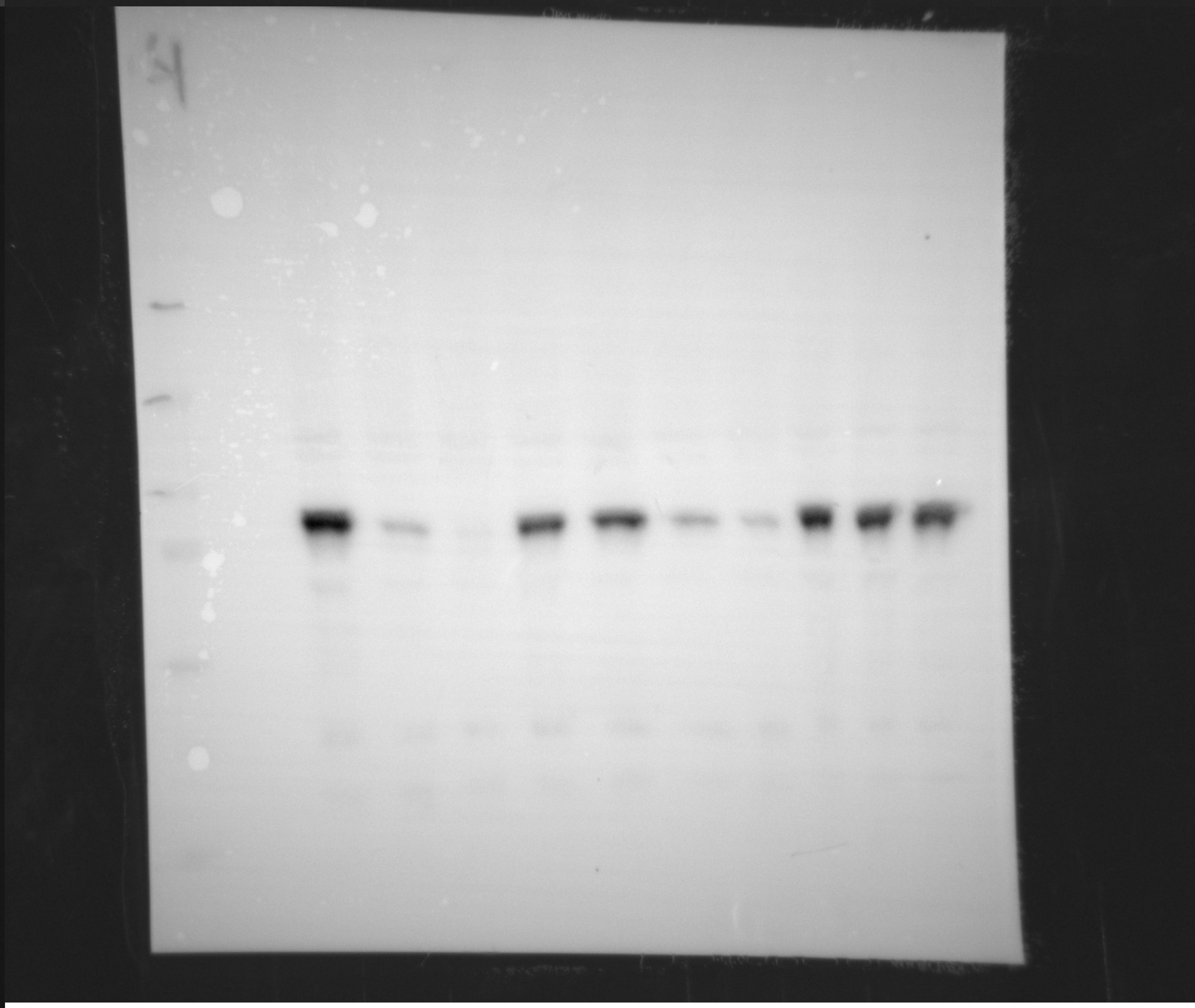

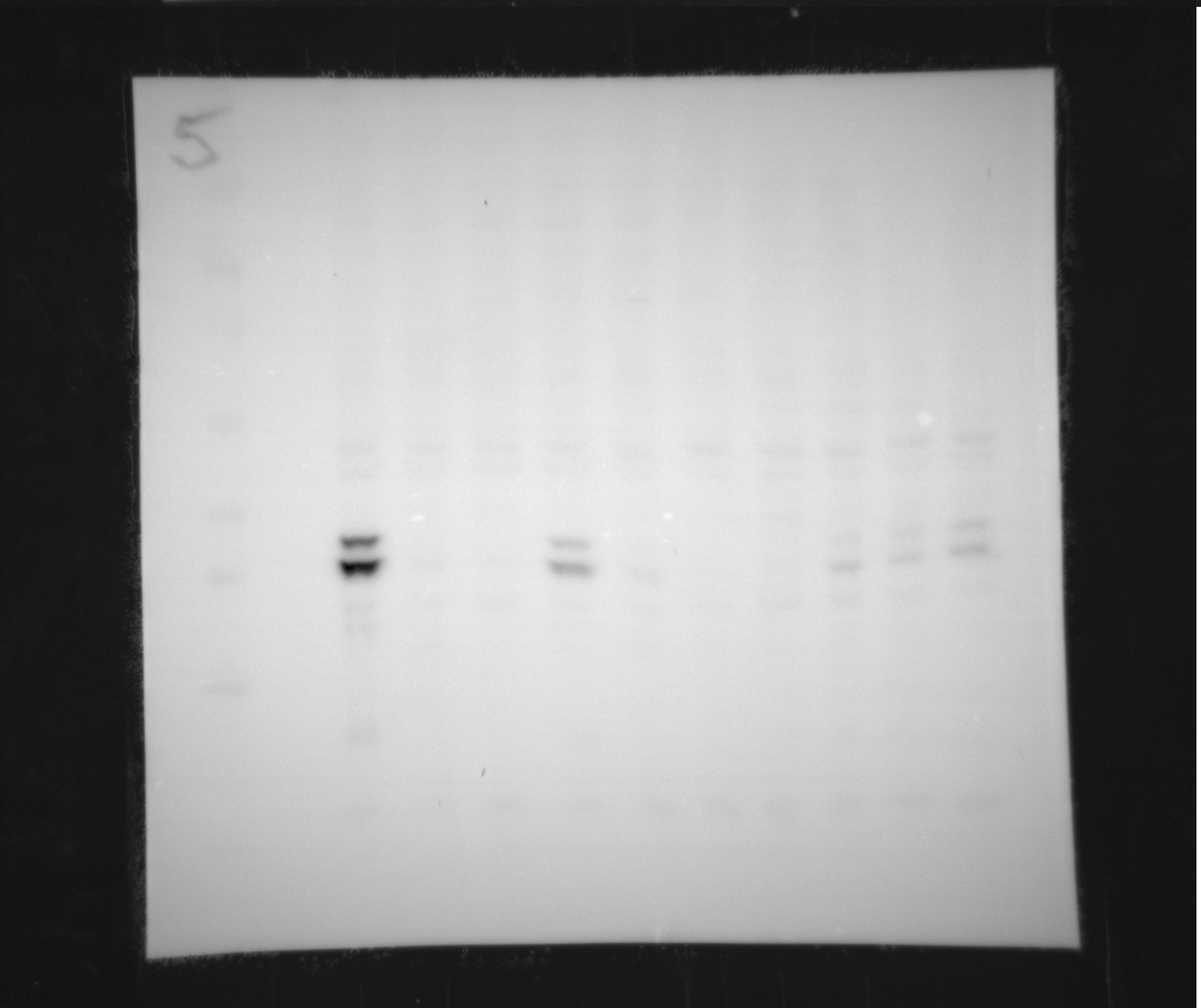

Blots:
1 (top left) - total MEK
2 (top right) - total ERK
3 (middle left) - total RSK
4 (middle right) - pMEK
5 (bottom left) - pERK

Lanes:
1 - DMSO
2 - Dabrafenib, 1 hour
3 - Dabrafenib, 30 minutes
4 - Dabrafenib, 15 minutes
5 - Trametinib, 15 minutes
6 - Trametinib, 30 minutes
7 - Trametinib, 1 hour
8 - SCH772984, 15 minutes
9 - SCH772984, 30 minutes
10 - SCH772984, 1 hour

Supplementary Figure S2

A

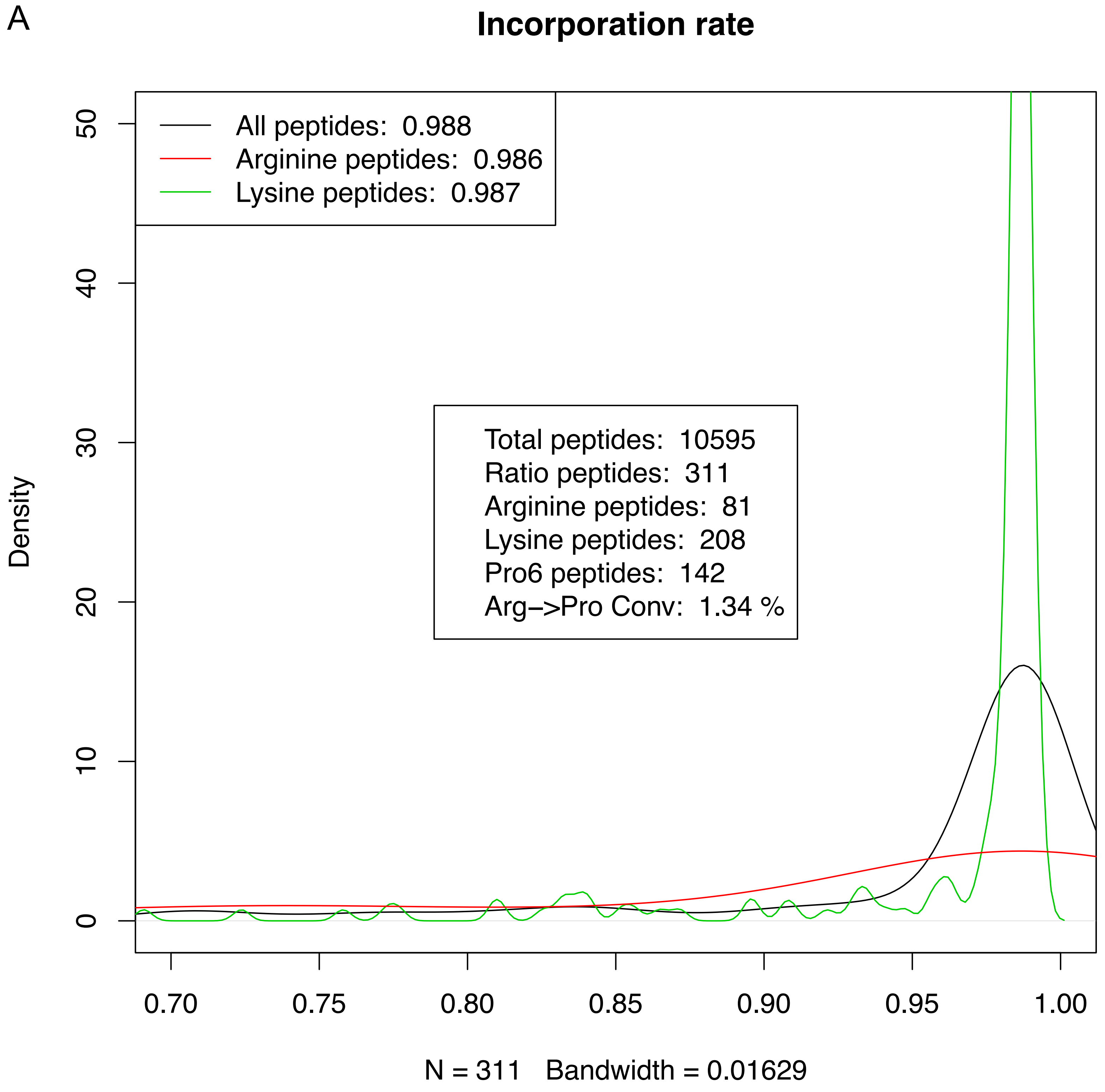

B

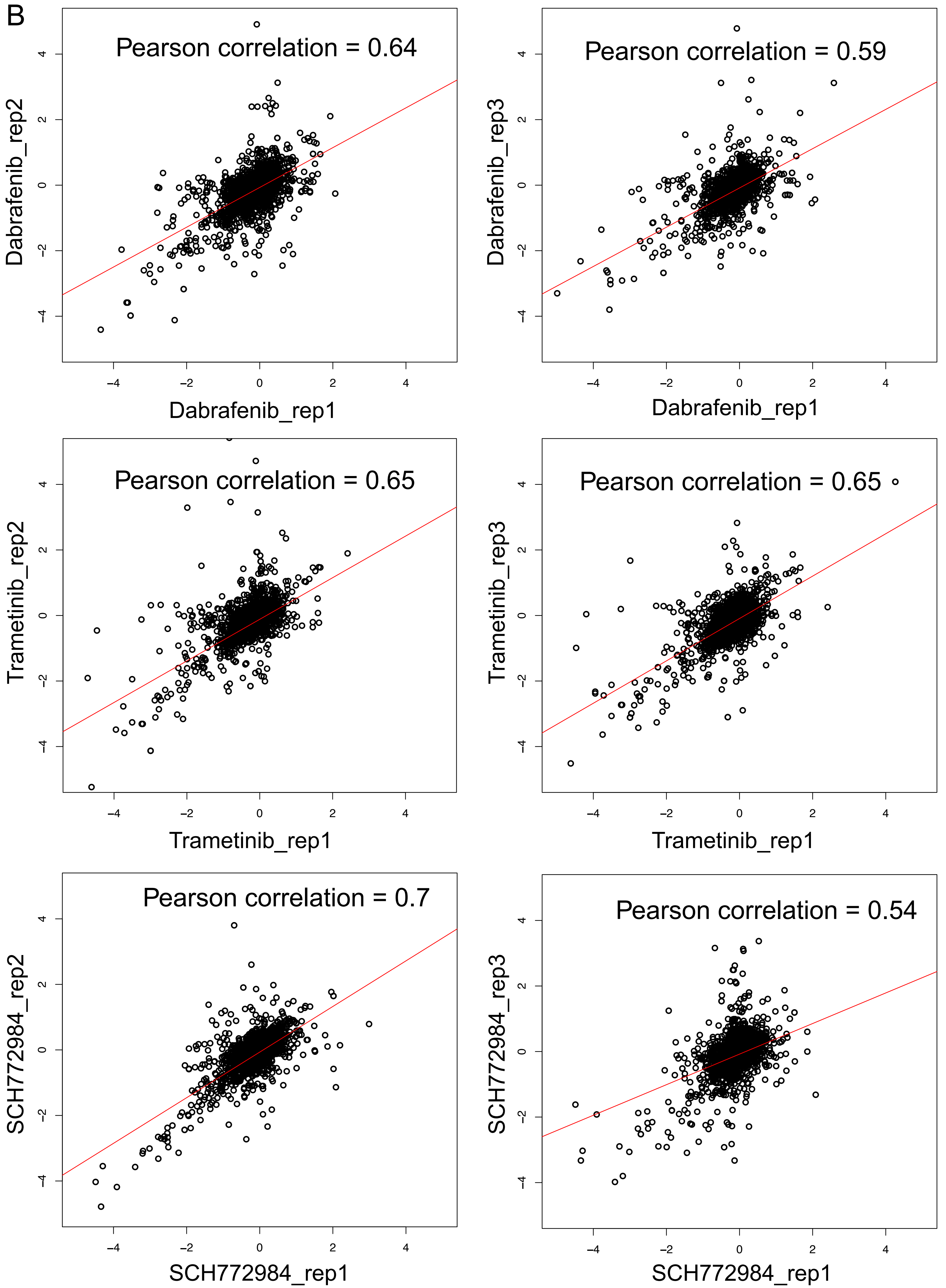

Supplementary Figure S3

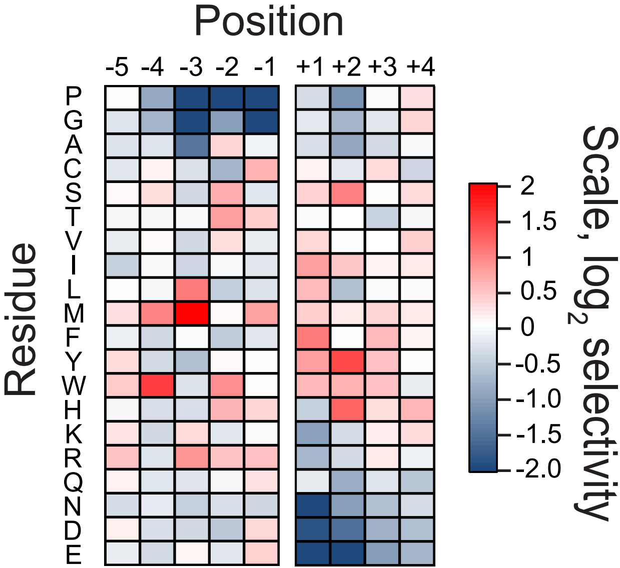

Supplementary Figure S4

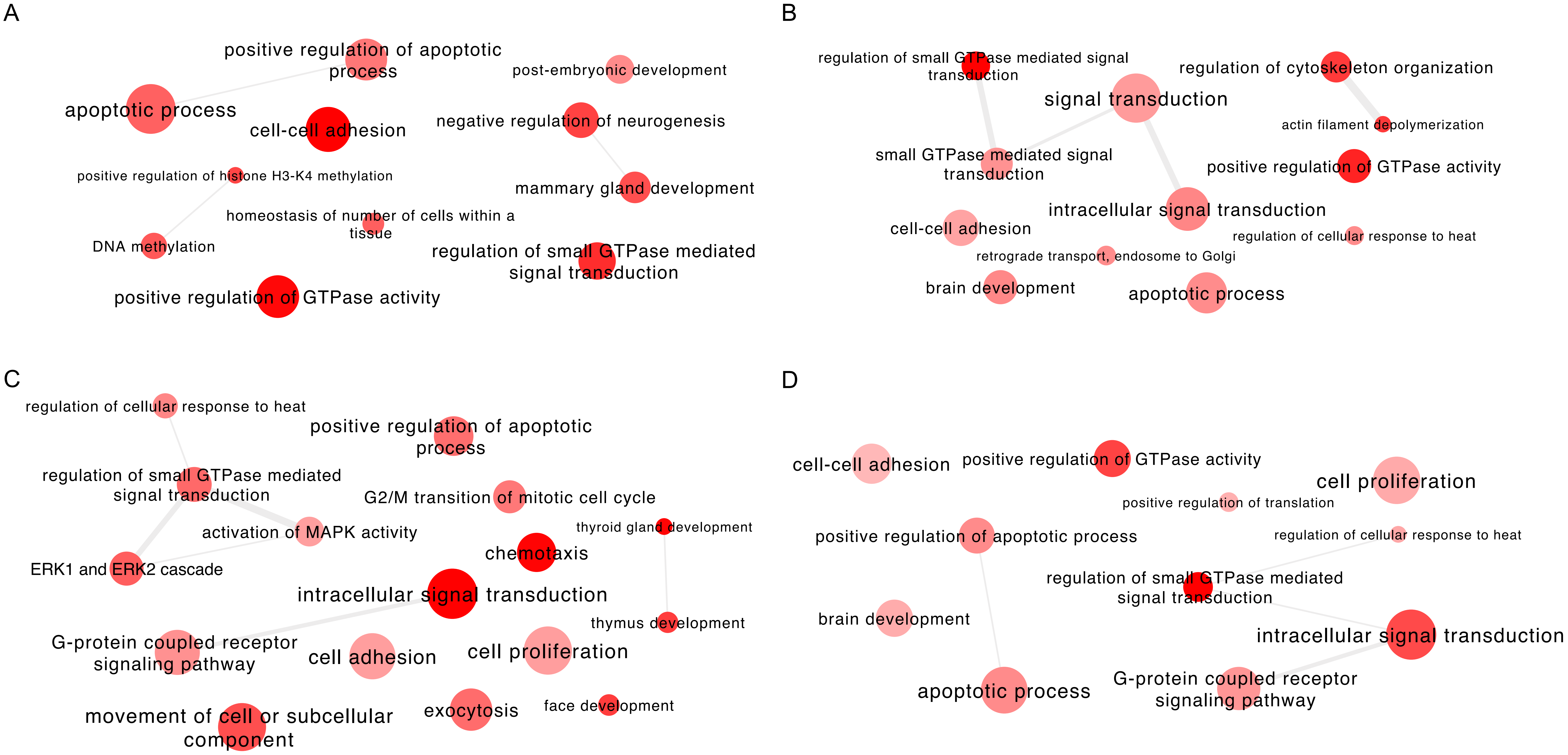

Supplement: Supplementary file 1 — Supplementary text and figures [file 41598_2019_47245_MOESM1_ESM.pdf]
